# Doped high-entropy glassy materials to create optical coherence from maximally disordered systems

**DOI:** 10.1038/s41377-022-00920-7

**Published:** 2022-07-18

**Authors:** Michele Marrocco

**Affiliations:** Department of Energy Technologies and Renewable Resources, ENEA (Italian National Agency for New Technologies, Energy and Sustainable Economic Development), Rome, Italy

**Keywords:** Solid-state lasers, Optical materials and structures

## Abstract

Control over excitation wavelengths, sample size, and doping concentrations in glassy materials with high levels of configuration entropy shows promises of efficient correlation between absorption and build-up of coherent emission of radiation.

More than 60 years ago, the first laser operation at the hands of Theodore H. Maiman^1^ unveiled the power of optical coherence (i.e., the fundamental feature of laser action) and gave birth to one of the most important scientific and technological revolutions in human history. Since then, uncountable applications and developments have been flooding the full extent of human activities. Lasers are found everywhere. Their popularity touches any fragment of our society. One example among many is the barcode reader which has become an indispensable tool for industrial and commercial purposes. Not to mention how the laser is an invaluable asset to science at large. However, more scientific and technological advancements are to come as though optical coherence was a wonder that never ceases to amaze us. Here, we present another innovation that holds the promise of more interesting applications. It regards the generation of optically coherent emission in materials where the entropic disorder nears its maximum.

The first question to ask is why should we care about materials of that kind? High-entropy materials (HEMs) are usually made of alloys^2–4^. They are very attractive for a number of reasons. To name them, good structural stability, high strength, and hardness, outstanding wear resistance, limited softening due to high temperatures, and low sensitivity to deterioration caused by corrosion and oxidation. These features result from the accurate design of such structures. To fulfill the objective, the trick resides in a random distribution of multiple atomic components filling the crystal lattice. They are also nearly equal in numbers and the final distribution determines a significant increase in the configuration entropy^5^. Under such a circumstance, being the relative abundance of such atomic components similar in space, the macroscopic picture is captured by a material where the evenly distributed disorder emulates a single-phase system^6^. Nonetheless, on the downside, these structures are not transparent and we cannot take advantage of their robust peculiarities when it comes to optical applications. Understandably, the optical regime requires the propagation of electromagnetic fields at visible or infrared wavelengths and opacity is detrimental to any ambitious goal of turning HEMs into novel optical media. The solution to the conundrum is within the reach of conventional laser physics and points towards glassy materials with optically active centers. Such systems mimic the optical medium used in known solid-state lasers (e.g., Nd:Glass^7^). The choice guarantees the successful realization of HEMs with great optical potentialities and, for obvious reasons, laser operation is captivating although challenging. The route is indeed arduous and presents itself with several unknowns.

A contribution aimed at exploring high-entropy glass systems (HEGSs) in view of laser operation comes from the work by Zhang et al.^8^. In their contribution, fundamental questions that HEGSs pose are explored and much emphasis is given to the role of phonon broadening in the coherent build-up. The phenomenon is rather intricate and needs some explaining. In glassy materials with multiple atomic components, vibrations (or phonons) propagating through the host deviate from the ideal quasi-monochromatic limit and vibrational energy is dispersed over many frequencies (phonon broadening). This manifestation is very common and carries information about thermal and transport characteristics of HEMs^9–11^. On the other hand, phonon broadening has seemingly an adverse effect when combined with the absorption and emission of radiation in ordinary materials. It represents a mechanical channel through which energy is dispersed and its unfavorable effect appears in the broadening of infrared absorption. On a more negative note, it must be underlined that spectral broadening is usually the precursor of incoherent optical phenomena and, as such, there is no correlation between absorption and emission. Instead, Zhang and collaborators find a novel and unexpected result: in HEGSs, there exists enough correlation to induce coherence. The outcome is really surprising and is complemented by the discovery that spatial coherence occurs at a low excitation threshold. How is this possible? The answer lies in the role played by phonon broadening.

The HEGS used by Zhang et al. is designed on the close packing of oxides. In particular, tetrahedral and octahedral voids are formed through close packing of O^2-^. The voids can be occupied by ions of different types and the presence of several atomic components widens the frequency of the collective vibrations. Therefore, high levels of configuration entropy in HEGSs imply inescapable phonon broadening. The energy exchange between spectrally broadened vibrational waves of the doped glassy medium and the excitation field is shown in Fig. 1 for the simplest case of one single incident wavelength (green). The simplification helps in identifying some of the fundamental processes expected for the generation of the broadened-phonon-assisted wideband radiation (BPAWR) and a subsequent self-absorption coherence modulation (SACM). In particular, BPAWR processes are responsible for blue- and red-shifted field components. They can have sufficient strength to trigger the cascade of stimulated emissions (i.e., the essential ingredient of laser action) that are at the core of the optical coherence found in HEGSs. This circumstance strongly depends on the phonon-assisted transitions that occur in both types of optical processes. The working principle can be summarized as follows. After excitation, a collective vibration removes (or add) part of the internal energy that has previously been absorbed. The subsequent radiative relaxation generates a field that starts to propagate (case b among the BPAWR processes of Fig. 1). This field initiates the build-up of stimulated radiation when the medium participates with excited states that are resonant with the propagating frequency (yellow). The condition occurs thanks to other combinations between spectrally broadened phonons and additional field components (cases f, g, and h among SACM processes of Fig. 1). Control over excitation wavelength, sample size, and doping concentration affects the SACM processes and, by playing with these control variables, Zhang and collaborators claim a good level of tunability so that the final emission can be tailored to the needs of specific purposes.

**Fig. 1:**
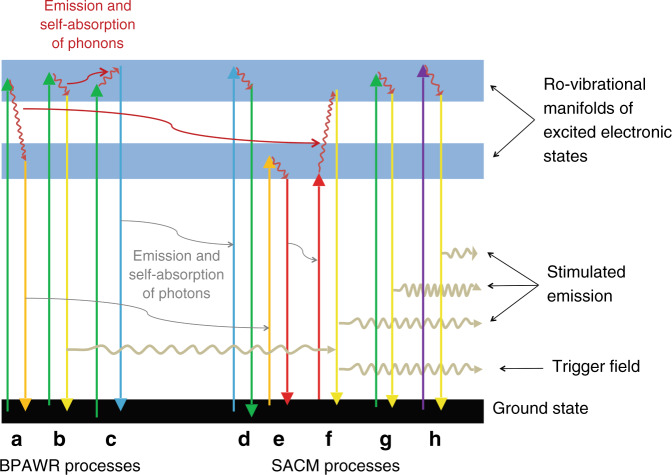
Schematic view of the photon- and phonon-assisted radiation generated by the BPAWR and SACM processes. The incident radiation is here represented by means of one single wavelength (green) for the sake of simplicity. BPAWR processes on the left side are responsible for blue- and red-shifted fields. They are generated after (**a**) excitation (green) and phonon propagation (dark red), (**b**) excitation (green) and a weaker phonon (dark red), and (**c**) excitation (green) in combination with an absorbed phonon (dark red). SACM processes relate to the previous ones by means of emission and self-absorption of photons (gray). Processes **f**, **g**, and **h** are responsible for the stimulated emission induced by the pre-existing trigger field generated within the BPAWR processes. Processes **d** and **e** are just other possible optical pathways among the many more not shown here

One limiting factor of the suggestion by Zhang et al. is the conversion efficiency which amounts to a fraction of %. This low conversion efficiency is due to the short mean free path of the phonons at room temperature. Their energy is turned into heat by the unavoidable phonon scattering and this constitutes an important limitation on the account of the strong dependence of SACM processes on phonon persistence. Therefore, better conversion values are expected at low temperatures that guarantee longer propagation distances of the vibrational wave.

Despite the limitation, the findings of Zhang and co-authors demonstrate a successful approach to fabricating novel HEMs with optical capabilities. While the potential of doped glassy materials for the generation of coherent radiation is well known^7^, the present work is free from the presence of an optical resonator that is conventionally used to boost the build-up of the coherent field. Furthermore, the extreme sensitivity to excitation wavelength, sample size, and doping concentration can be used to engineer spectrally broad emissions. Hence, the proposed research is of inspiration to make more significant contributions to the development of a light source made of HEMs with a high degree of optical coherence spanning a large portion of the spectrum. The availability of broadband and widely tunable systems is indeed important for the innovative research areas of supercontinuum generation, laser amplification, and advanced optical communication.
